# Supplementary material for: Regional nonsense constraint offers biological and clinical insights into genetic disease
Source: Nat Commun. 2026 Feb 25;17:3152. doi: 10.1038/s41467-026-69983-z (PMC13043745; doi:10.1038/s41467-026-69983-z)
Supplement: Supplementary file 1 — Supplementary Information [file 41467_2026_69983_MOESM1_ESM.pdf]

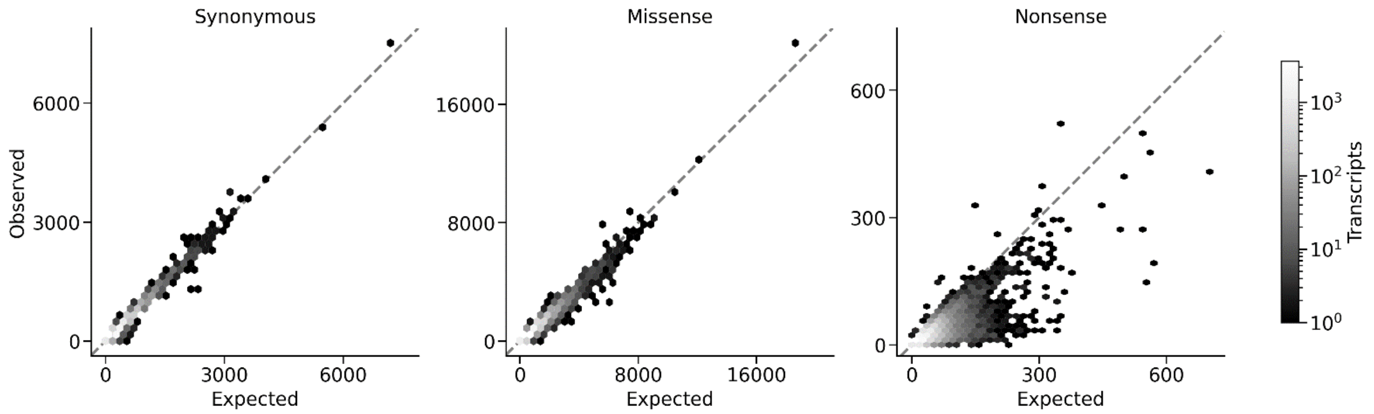

Supplementary Figure 1| Modelling the expected number of synonymous, missense, and nonsense variants with Roulette. Hexagonal binned plots comparing the number of SNVs observed in gnomAD v4.1 versus the number expected by the null mutational model based on Roulette for 19,676 canonical protein-coding transcripts (Methods). The shading of the hexagons corresponds to the number of transcripts in each bin. The dashed grey line marks x=y. For visual clarity, *TTN* (ENST00000589042) is not plotted. Source data are provided as a Source Data file.

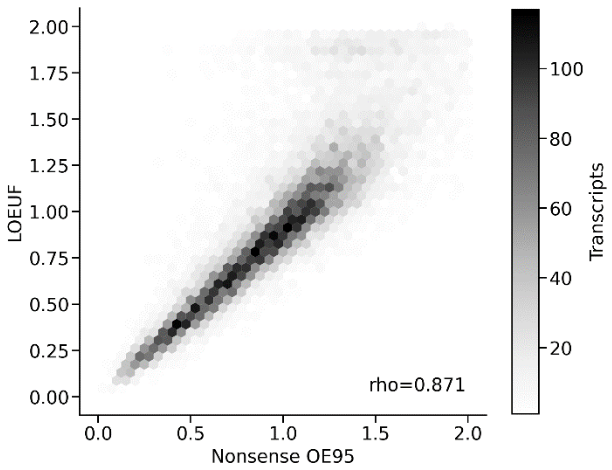

Supplementary Figure 2| Hexagonal binned plot showing transcript-level nonsense OE95 versus LOEUF. The shading of each hexagon corresponds to the number of transcripts within that bin. Spearman's rank correlation coefficient (rho) is shown. Scores greater than 2 are not plotted for visual clarity. Source data are provided as a Source Data file.

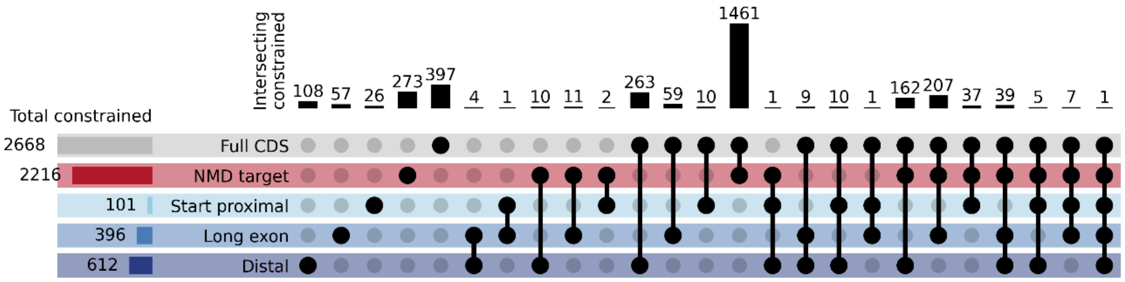

Supplementary Figure 3| Upset plot showing the number of transcripts with regional nonsense constraint. Horizontal bars show the number of transcripts with nonsense constraint in each region. Vertical bars show the number of transcripts which harbour one or more constrained regions. Intersecting regions are shown in the matrix. For example, 273 transcripts are constrained only in NMD target regions (fourth column from left).

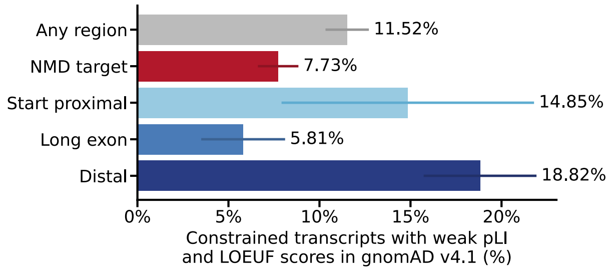

Supplementary Figure 4| The percentage of transcripts with regional nonsense constraint which have weak pLI/LOEUF scores in gnomAD v4.1. The precise number of variants in each category are shown in Supplementary Figure 5. Error bars show 95% confidence intervals. Source data are provided as a Source Data file.

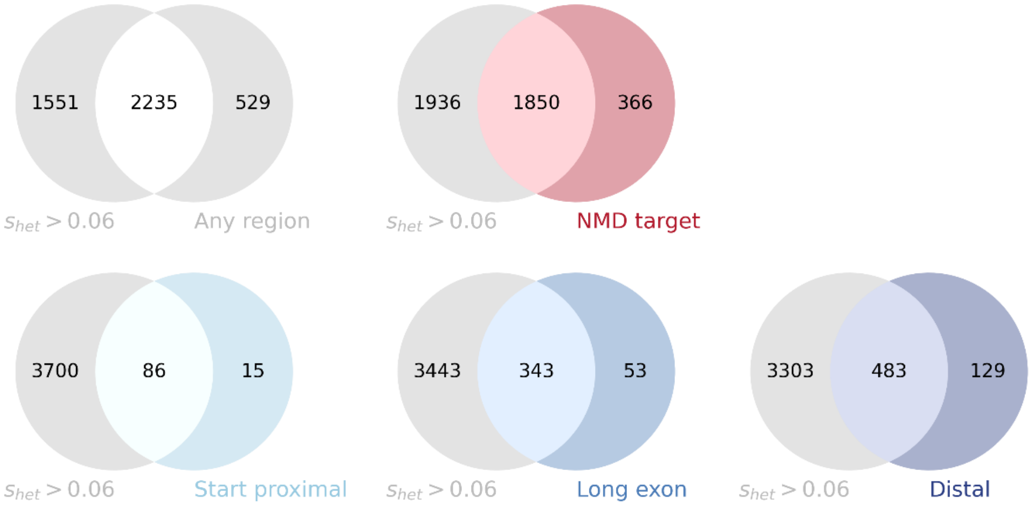

Supplementary Figure 5| Comparison of genes prioritised by GeneBayes and regional nonsense constraint. Venn diagrams show genes constrained by GeneBayes only (s_{het}>0.06) (leftmost set), GeneBayes and regional nonsense constraint (intersection) or by regional nonsense constraint only (rightmost set). Constraint scores were taken for the canonical transcript of each gene (see Methods). The s_{het} cutoff of 0.06 corresponds approximately to a LOEUF score of 0.6, and is more conservative than the suggested cutoff of s_{het} 0.1, which corresponds to a LOEUF score of 0.35. Source data are provided as a Source Data file.

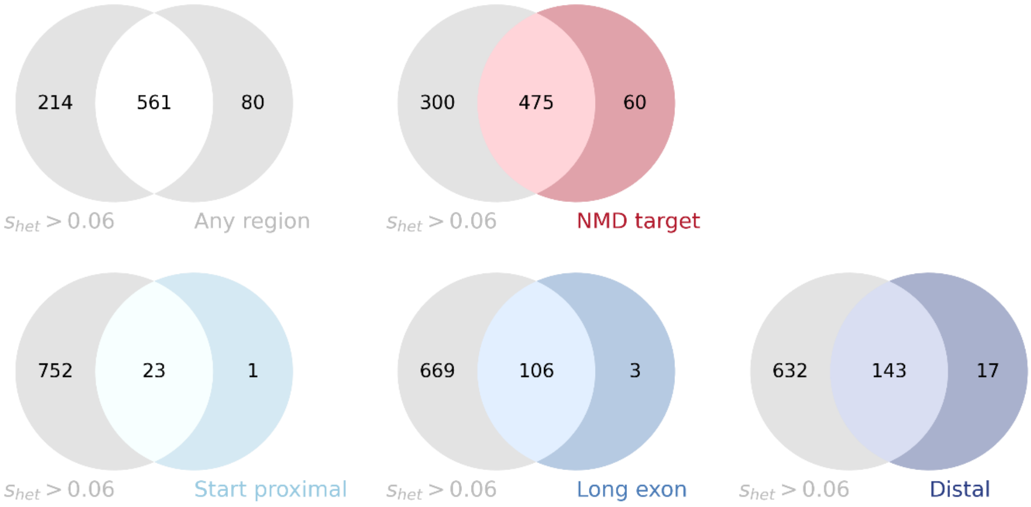

Supplementary Figure 6| Comparison of genes with an autosomal dominant disease association in OMIM, prioritised by GeneBayes and regional nonsense constraint. Venn diagrams show genes constrained by GeneBayes only (s_{het}>0.06) (leftmost set), GeneBayes and regional nonsense constraint (intersection) or by regional nonsense constraint only (rightmost set). Constraint scores were taken for the canonical transcript of each gene (see Methods). The s_{het} cutoff of 0.06 corresponds approximately to a LOEUF score of 0.6, and is more conservative than the suggested cutoff of s_{het} 0.1, which corresponds to a LOEUF score of 0.35. Source data are provided as a Source Data file.

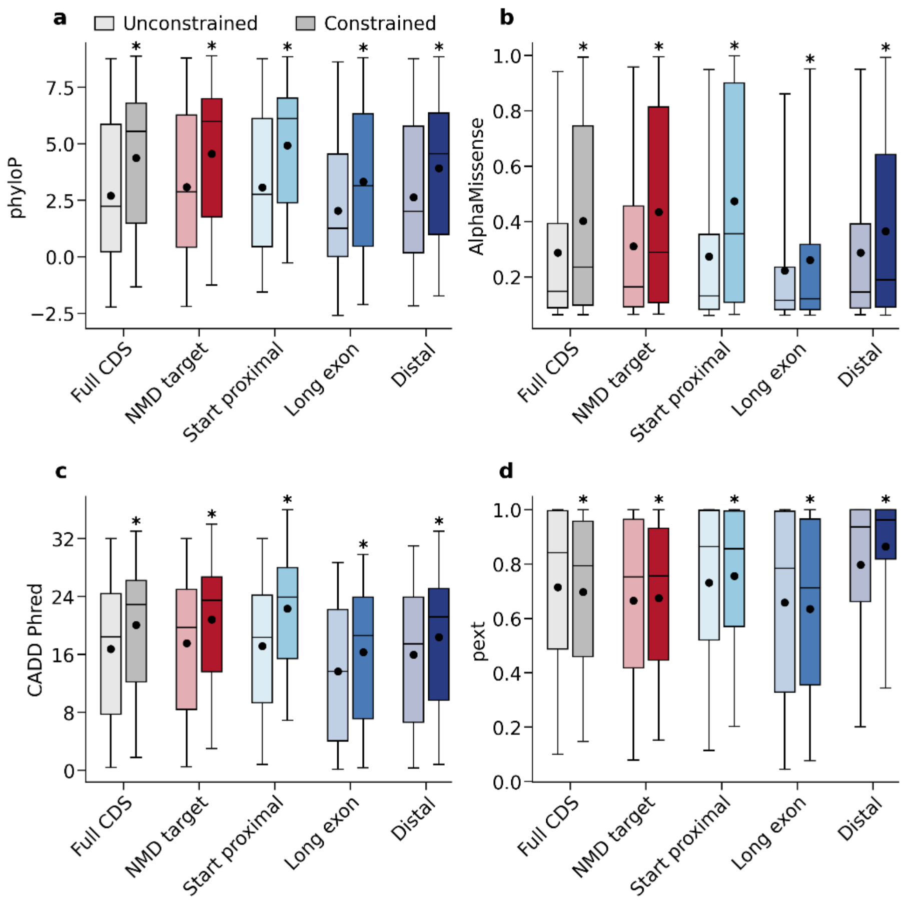

Supplementary Figure 7| Conservation, pathogenicity prediction, and expression of sites within constrained and unconstrained regions. NMD-target regions are shown in red, NMD-escape regions in blue, and full coding sequence (CDS) in grey. Whiskers show the 5th and 95th percentiles, the box shows the 25th and 75th percentiles, and the horizontal line shows the 50th percentile. Closed circles show the mean. Asterisks indicate mean scores which are significantly different in constrained versus unconstrained regions (two-sided Welch's T tests, with Bonferroni correction for 20 tests at alpha=0.05, $P<10^{-8}$) **a)** Mean phyloP scores. **b)** Mean AlphaMissense scores. **c)** Mean CADD Phred scores. **d)** Mean pext scores. Source data are provided as a Source Data file.

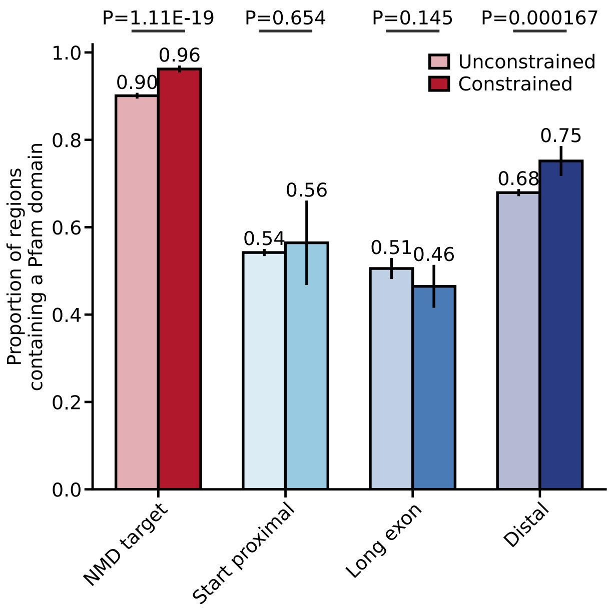

Supplementary Figure 8| Pfam domains in NMD regions. Bar chart showing the proportion of NMD regions containing a Pfam domain. NMD-target regions are shown in red, NMD-escape regions in blue. Paired bars show unconstrained regions (lighter fill) and constrained regions (darker fill). Error bars show 95% confidence intervals. P values for two-tailed Z tests of proportion are shown. Source data are provided as a Source Data file.

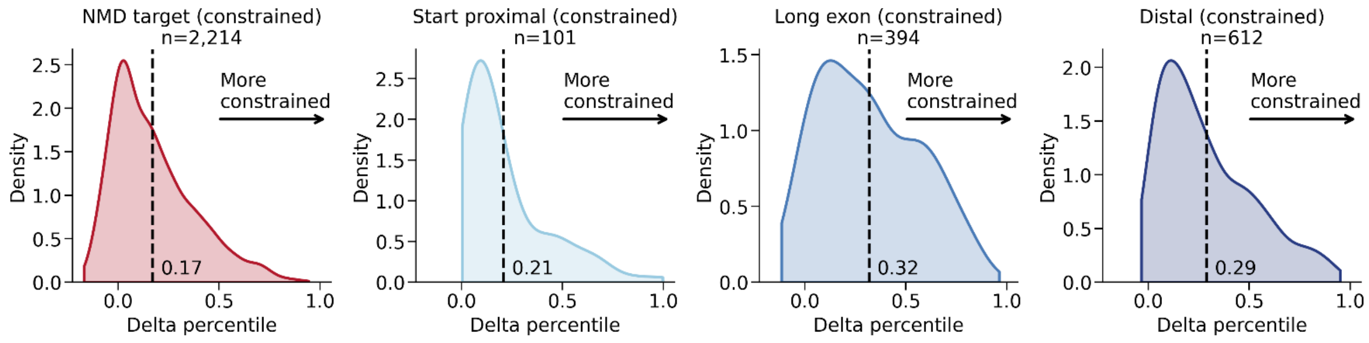

Supplementary Figure 9| Discrepant between-species conservation and within-species constraint in constrained regions. Kernel density estimation for the percentile difference between OE95 scores and basewise phyloP scores in constrained regions. The dashed black line shows the mean percentile difference. The number of genes constrained in each region is shown. Source data are provided as a Source Data file.

Region	Constraint	O/E	O/E upper 95% CI	pLI	LOEUF
**Transcript**	Constrained	0.33	0.53	0.95	0.53
**NMD target**	Constrained	0	0.19		
**Start-proximal**	Unconstrained	1.6	3.4		
**Long exon**	Unconstrained	0.60	1.0		
**Distal**	NA	0.21	0.90		

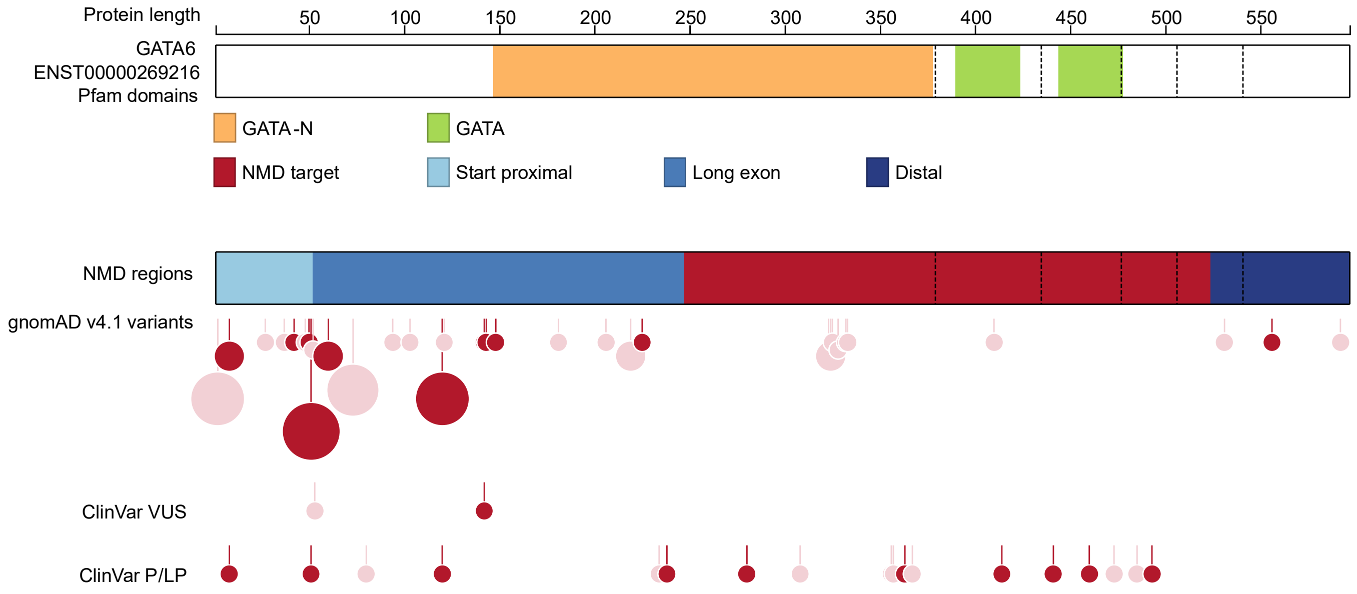

Supplementary Figure 10| Nonsense variants are unconstrained in the start-proximal and long exon NMD-escape regions of *GATA6* (ENST00000269216), as previously described[22]. This and the other Supplementary Figures are arranged as in Figure 3a.

Region	Constraint	O/E	O/E upper 95% CI	pLI	LOEUF
**Transcript**	NA	0.59	0.89	0.01	0.75
**NMD target**	Constrained	0.19	0.58		
**Start-proximal**	Unconstrained	0.25	1.1		
**Long exon**	NA	NA	NA		
**Distal**	Unconstrained	1.2	1.8		

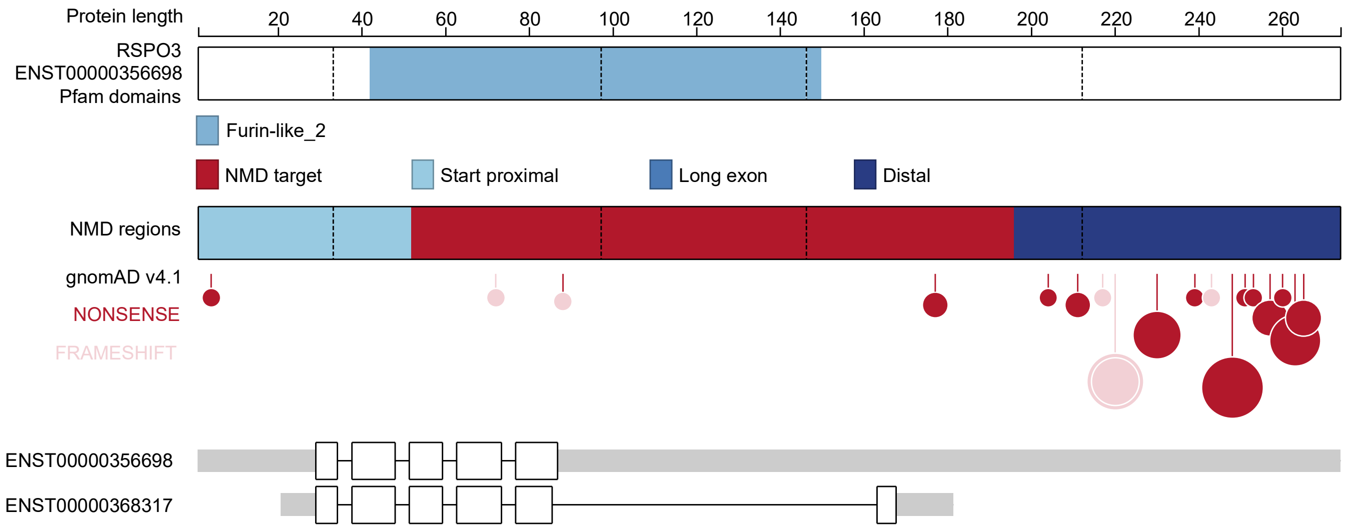

Supplementary Figure 11| Despite weak pLI and LOEUF scores, the NMD-target region of *RSPO3* (ENST00000356698) is strongly constrained for nonsense variants.

Region	Constraint	O/E	O/E upper 95% CI	pLI	LOEUF
**Transcript**	NA	0.60	0.96	0.04	0.77
**NMD target**	Unconstrained	0.76	1.4		
**Start-proximal**	Constrained	0	0.57		
**Long exon**	NA	NA	NA		
**Distal**	Unconstrained	0.92	1.7		

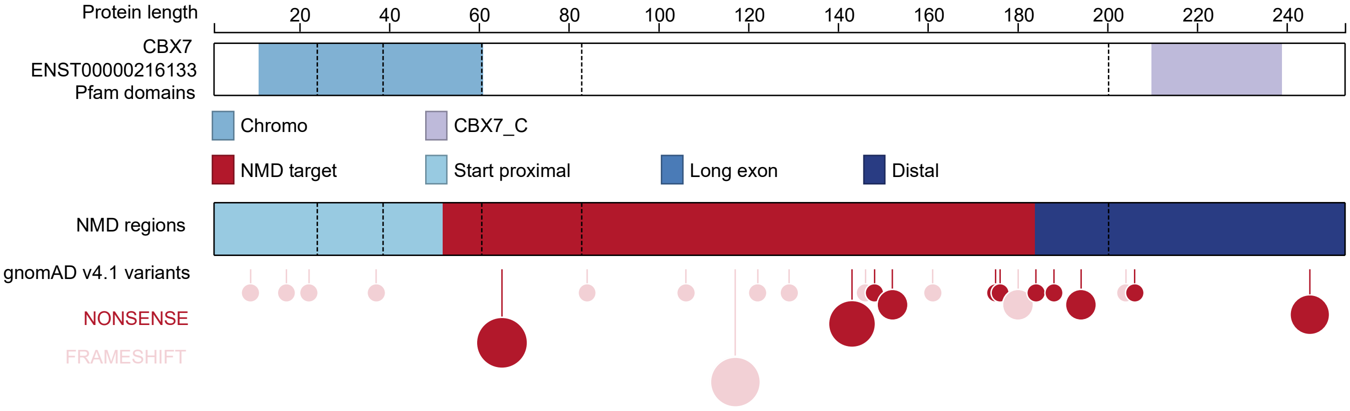

Supplementary Figure 12| Start-proximal constraint in *CBX7* (ENST00000216133).

Region	Constraint	O/E	O/E upper 95% CI	pLI	LOEUF
**Transcript**	NA	0.55	0.77	0.60	0.60
**NMD target**	NA	0.54	0.89		
**Start-proximal**	Unconstrained	1.3	2.4		
**Long exon**	Constrained	0.15	0.47		
**Distal**	Unconstrained	0.93	1.6		

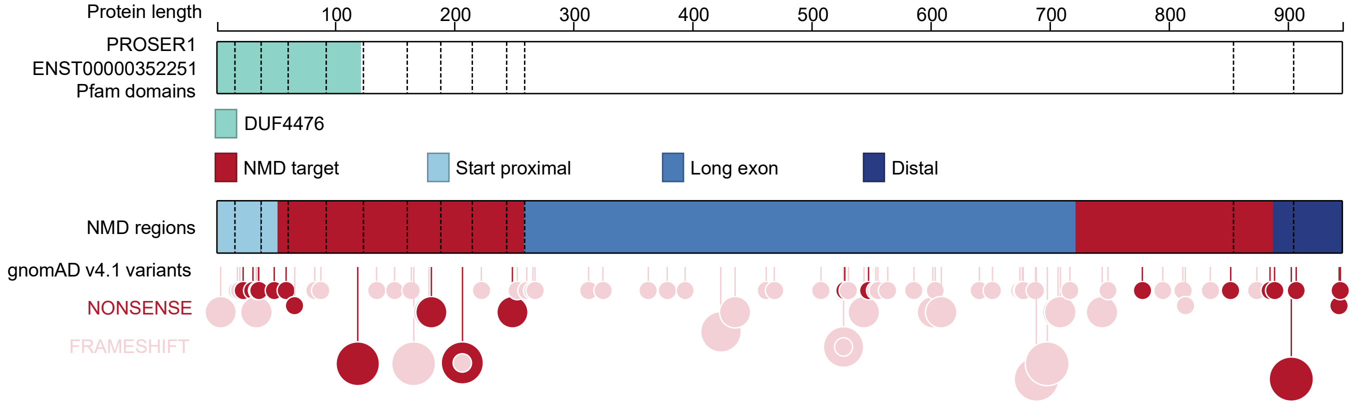

Supplementary Figure 13| Nonsense variants are highly constrained in the long exon NMD-escape region of *PROSER1* (ENST00000352251). The bimodal distribution of nonsense variants contrasts with the more uniform distribution of frameshift variants.

Region	Constraint	O/E	O/E upper 95% CI	pLI	LOEUF
**Transcript**	Constrained	0.29	0.51	0.41	0.71
**NMD target**	NA	0.19	0.85		
**Start-proximal**	Unconstrained	0.66	1.5		
**Long exon**	Unconstrained	0.58	1.2		
**Distal**	Constrained	0	0.26		

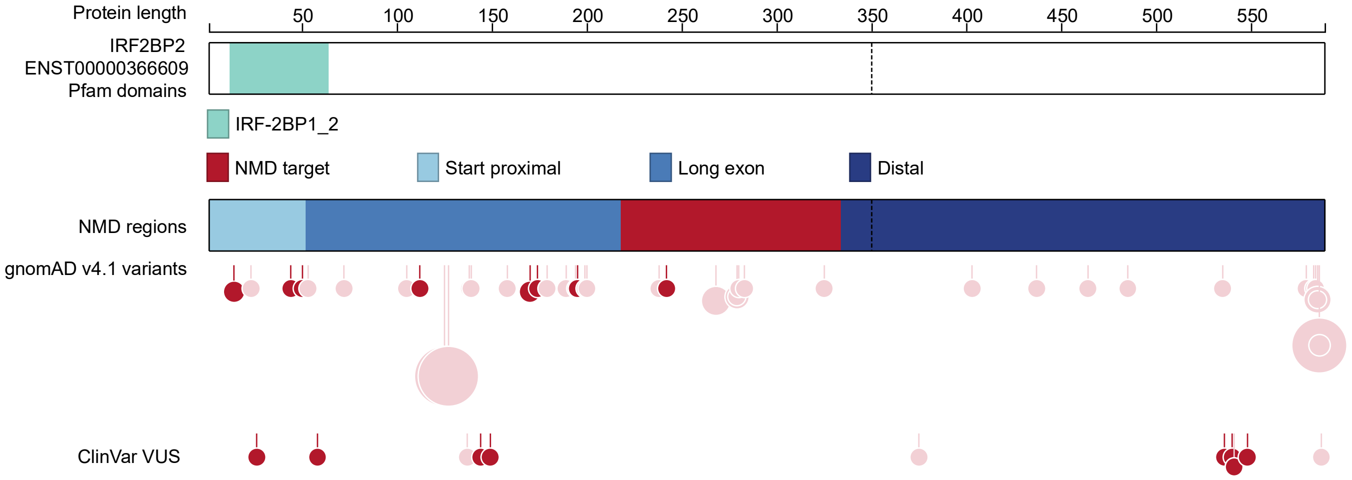

Supplementary Figure 14| Nonsense variants are highly constrained in the distal NMD-escape region of *IRF2BP2* (ENST00000366609), but not in the 5' CDS.

Region	Constraint	O/E	O/E upper 95% CI	pLI	LOEUF
**Transcript**	NA	0.75	0.97	0	0.85
**NMD target**	Unconstrained	0.57	1.1		
**Start-proximal**	Unconstrained	1.4	2.6		
**Long exon**	Unconstrained	0.91	1.2		
**Distal**	Constrained	0	0.45		

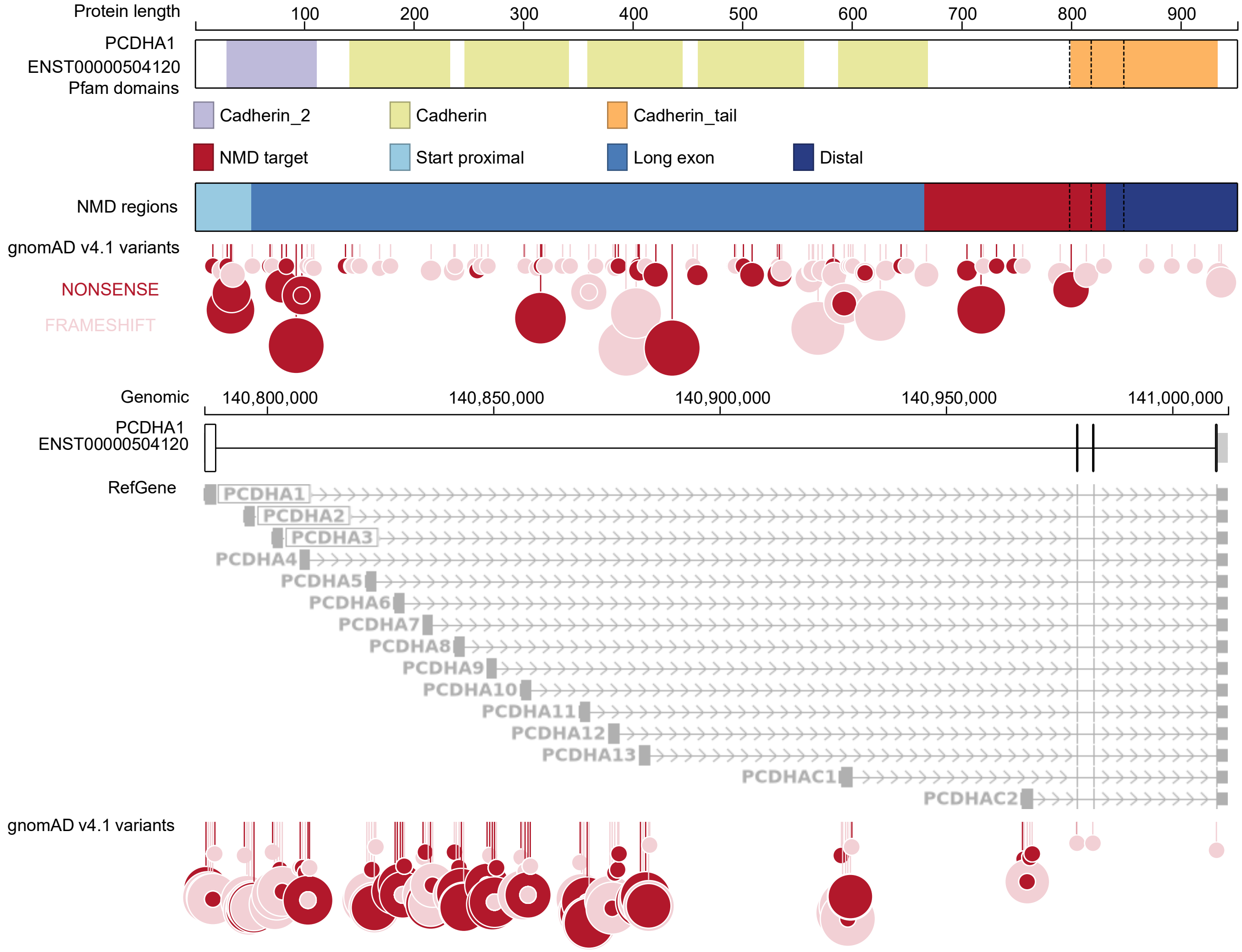

Supplementary Figure 15| Genes in the protocadherin alpha cluster have strong nonsense constraint in the distal NMD-escape regions. The final three exons, encoding a conserved cytoplasmic domain, are shared by all genes in the cluster.

Region	Constraint	O/E	O/E upper 95% CI	pLI	LOEUF
Transcript	NA	0.68	0.90	0	0.71
NMD target	Unconstrained	0.67	1.2		
Start-proximal	Unconstrained	0.58	2.5		
Long exon	NA	0.72	1.0		
Distal	Unconstrained	0.48	1.4		

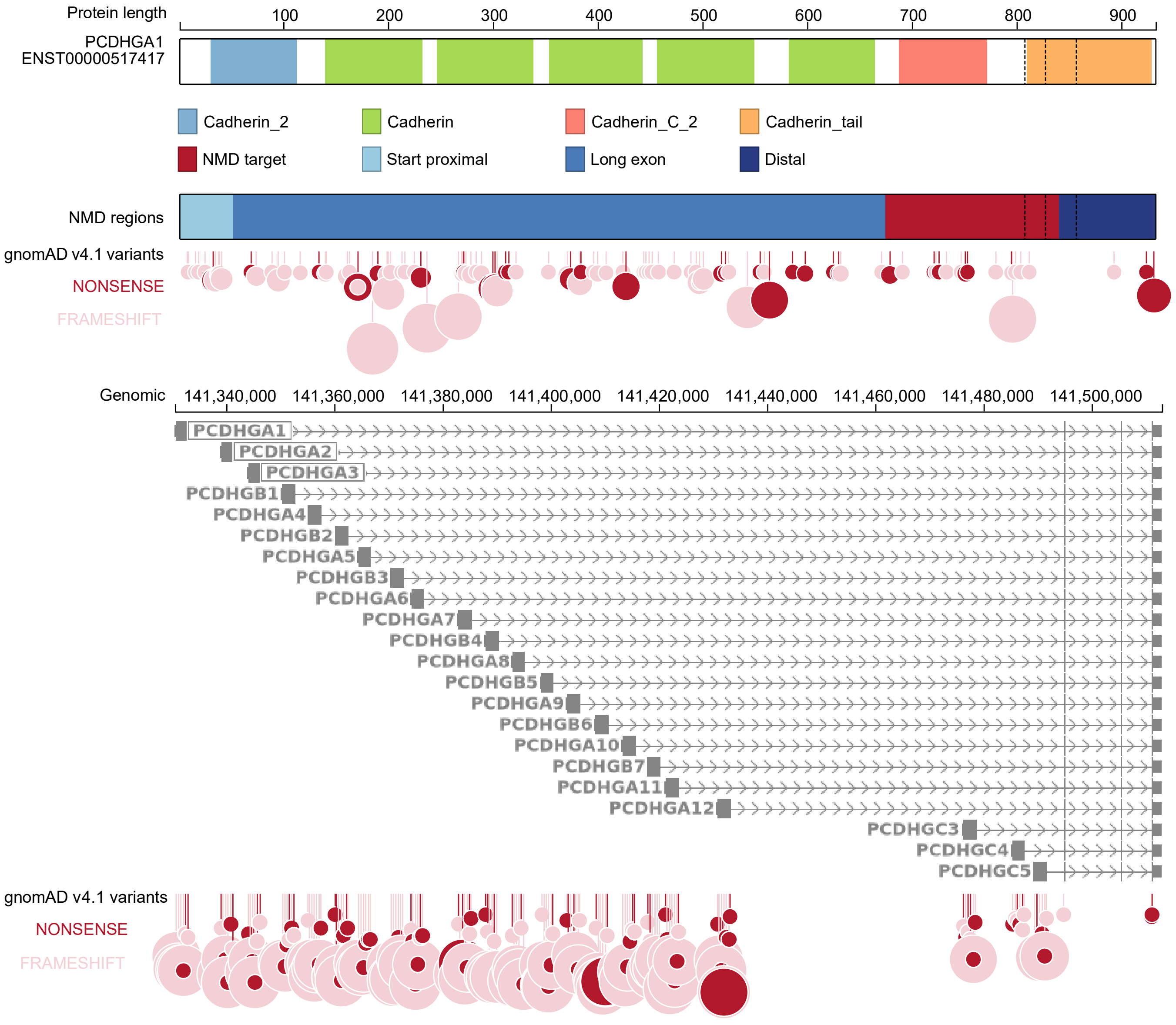

Supplementary Figure 16| The distribution of gnomAD v4.1 nonsense variants in the protocadherin gamma cluster.

Region	Constraint	O/E	O/E upper 95% CI	pLI	LOEUF
**Transcript**	Constrained	0.20	0.27	1.0	0.23
**NMD target**	NA	0.45	0.67		
**Start-proximal**	Unconstrained	0.63	1.80		
**Long exon**	Constrained	0.04	0.17		
**Distal**	Constrained	0.12	0.21		

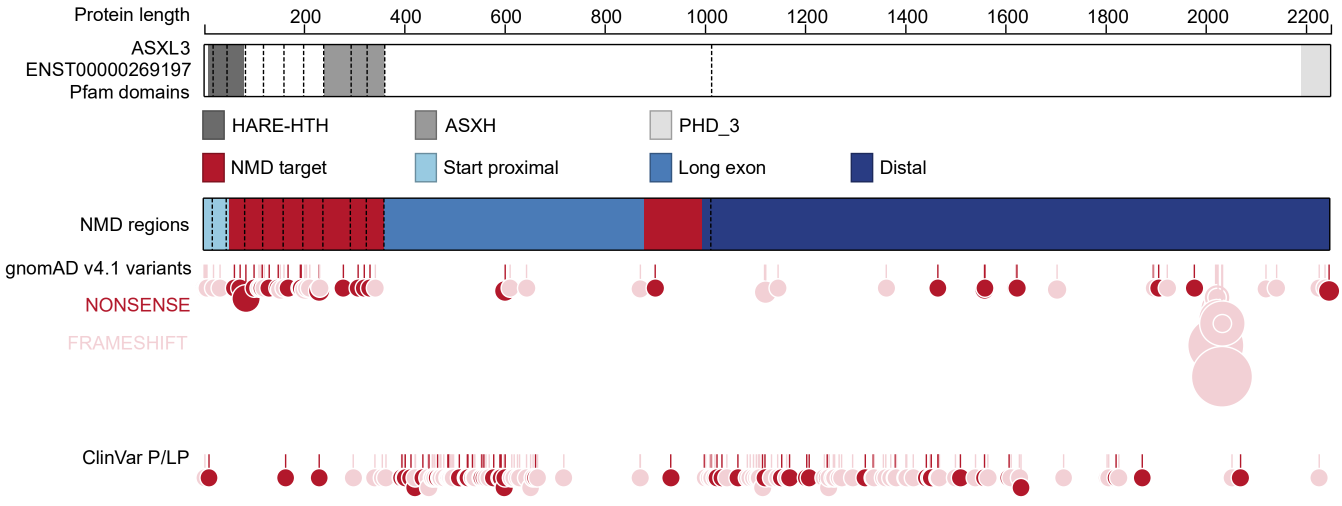

Supplementary Figure 17| Nonsense variants are strongly constrained in the long exon and distal NMD-escape regions of *ASXL3* (ENST00000269197), whereas the NMD-target region is relatively tolerant to PTCs. Pathogenic nonsense and frameshift variants in ClinVar cluster in the constrained regions.

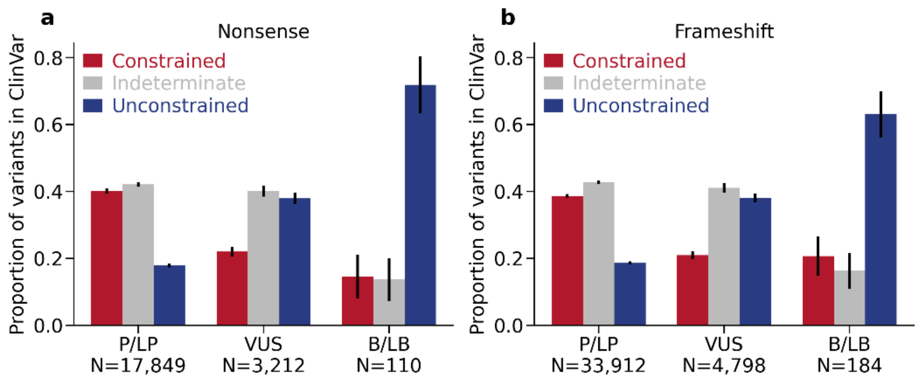

Supplementary Figure 18| Proportion of ClinVar variants in known autosomal dominant disease genes falling in constrained, indeterminate, or unconstrained regions. The data are stratified by ACMG variant classification. **a)** Nonsense variants. **b)** Frameshift variants. P/LP = Pathogenic / Likely Pathogenic, VUS = Variant of Uncertain Significance, B/LB = Benign / Likely Benign. Source data are provided as a Source Data file.

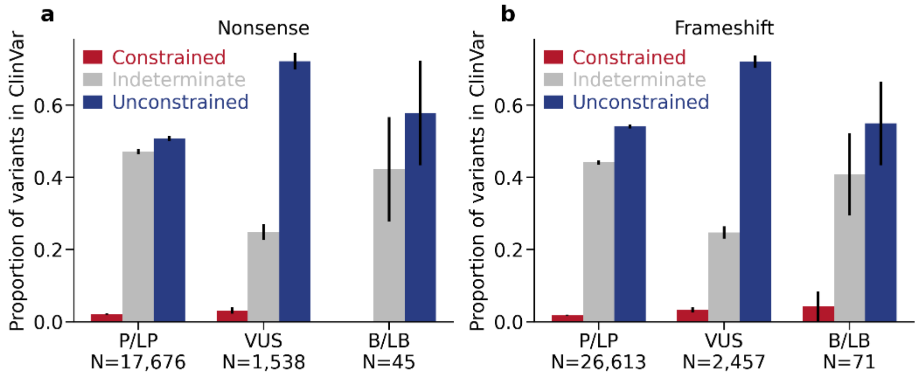

Supplementary Figure 19| Proportion of ClinVar variants in known autosomal recessive disease genes falling in constrained, indeterminate, or unconstrained regions. The data are stratified by ACMG variant classification. **a)** Nonsense variants. **b)** Frameshift variants. P/LP = Pathogenic / Likely Pathogenic, VUS = Variant of Uncertain Significance, B/LB = Benign / Likely Benign. Source data are provided as a Source Data file.
