# Supplementary material for: Regional nonsense constraint offers biological and clinical insights into genetic disease
Source: Nat Commun. 2026 Feb 25;17:3152. doi: 10.1038/s41467-026-69983-z (PMC13043745; doi:10.1038/s41467-026-69983-z)
Supplement: Supplementary file 2 — Description of Additional Supplementary Files [file 41467_2026_69983_MOESM2_ESM.pdf]

Description of Additional Supplementary Files

Supplementary Data 1| 21 genes with extreme divergence in regional nonsense constraint (OE95 >90th centile) and basewise conservation (median phyloP <90th centile) scores.

Supplementary Data 2| Longlist of candidate disease genes which have no association with an autosomal dominant phenotype in OMIM, and carry at least 3 *de novo* nonsense or frameshift variants in a constrained NMD region in 27,762 rare disease trios.

Supplementary Data 3| Regional nonsense constraint annotations and summary statistics.
