# Supplementary material for: Regional nonsense constraint offers biological and clinical insights into genetic disease
Source: Nat Commun. 2026 Feb 25;17:3152. doi: 10.1038/s41467-026-69983-z (PMC13043745; doi:10.1038/s41467-026-69983-z)
Supplement: Supplementary file 6 — Reporting Summary [file 41467_2026_69983_MOESM6_ESM.pdf]

nature portfolio

Corresponding author(s): Siddharth Banka

Last updated by author(s): Jan 21, 2026

Reporting Summary

Nature Portfolio wishes to improve the reproducibility of the work that we publish. This form provides structure for consistency and transparency in reporting. For further information on Nature Portfolio policies, see our Editorial Policies and the Editorial Policy Checklist.

Statistics

For all statistical analyses, confirm that the following items are present in the figure legend, table legend, main text, or Methods section.

n/a	Confirmed	
☐	☒	The exact sample size (*n*) for each experimental group/condition, given as a discrete number and unit of measurement
☐	☒	A statement on whether measurements were taken from distinct samples or whether the same sample was measured repeatedly
☐	☒	The statistical test(s) used AND whether they are one- or two-sided<br>*Only common tests should be described solely by name; describe more complex techniques in the Methods section.*
☒	☐	A description of all covariates tested
☐	☒	A description of any assumptions or corrections, such as tests of normality and adjustment for multiple comparisons
☐	☒	A full description of the statistical parameters including central tendency (e.g. means) or other basic estimates (e.g. regression coefficient) AND variation (e.g. standard deviation) or associated estimates of uncertainty (e.g. confidence intervals)
☐	☒	For null hypothesis testing, the test statistic (e.g. *F*, *t*, *r*) with confidence intervals, effect sizes, degrees of freedom and *P* value noted<br>*Give P values as exact values whenever suitable.*
☒	☐	For Bayesian analysis, information on the choice of priors and Markov chain Monte Carlo settings
☒	☐	For hierarchical and complex designs, identification of the appropriate level for tests and full reporting of outcomes
☒	☐	Estimates of effect sizes (e.g. Cohen's *d*, Pearson's *r*), indicating how they were calculated

Our web collection on statistics for biologists contains articles on many of the points above.

Software and code

Policy information about availability of computer code

Data collection	No software was used in the collection of data for this study.
Data analysis	The following software was used in the analysis of data for this study: Python 3.8.17 (a full list of dependencies and Python libraries used in this study are shared in our publicly available Github repository), bedtools v2.31.1, BCFtools v1.20, Variant Effect Predictor (VEP) v105, Biorender.

For manuscripts utilizing custom algorithms or software that are central to the research but not yet described in published literature, software must be made available to editors and reviewers. We strongly encourage code deposition in a community repository (e.g. GitHub). See the Nature Portfolio guidelines for submitting code & software for further information.

Data

Policy information about availability of data

All manuscripts must include a data availability statement. This statement should provide the following information, where applicable:

- Accession codes, unique identifiers, or web links for publicly available datasets
- A description of any restrictions on data availability
- For clinical datasets or third party data, please ensure that the statement adheres to our policy

Regional nonsense constraint annotations and summary statistics are available in Supplementary Dataset 3, and for download at https://github.com/alexblakes/regional_nonsense_constraint/blob/main/data/final/regional_nonsense_constraint.tsv. Regional constraint annotations are also publicly available through the DECIPHER portal [URL to be added]. Source data are provided with this paper. For Figure 6, export of Source Data from the Genomics England Research

Environment is not permitted. These data are available to registered users of the National Genomic Research Library at /re_gecip/shared_allGeCIPs/AlexBlakes/regional_nonsense_constraint/figure_6.tsv.
Data from the National Genomic Research Library (NGRL) used in this research are available within the secure Genomics England Research Environment. Access to NGRL data is restricted to adhere to consent requirements and protect participant privacy.
Access to NGRL data is provided to approved researchers who are members of the Genomics England Research Network, subject to institutional access agreements and research project approval under participant-led governance. For more information on data access, visit: https://www.genomicsengland.co.uk/research

Research involving human participants, their data, or biological material

Policy information about studies with human participants or human data. See also policy information about sex, gender (identity/presentation), and sexual orientation and race, ethnicity and racism.

Reporting on sex and gender	No sex- or gender-based analyses were performed.
Reporting on race, ethnicity, or other socially relevant groupings	No analyses based on race, ethnicity, or other groupings were performed.
Population characteristics	This study used sequencing data from participants in:<br>- The gnomAD study v4.1 (https://gnomad.broadinstitute.org/about)<br>- Rare disease cohorts, including:<br>- - Individuals recruited to the 100,000 Genomes Project<br>- - Individuals recruited to the NHS Genomic Medicine Service<br>- - Individuals recruited to sequencing studies through the Deciphering Developmental Disorders study, GeneDx, and Radboud University Medical Center.
Recruitment	Participants in the rare disease cohorts were recruited on the basis that they or a family member were affected by a rare, presumably genetic, condition or developmental disorder.
Ethics oversight	The aggregation and release of summary data from the exomes and genomes collected by the Genome Aggregation Database has been approved by the Mass General Brigham IRB (protocol 2013P001339, "Large-scale aggregation of human genomic data").<br>Genomics England has approval from the HRA Committee East of England – Cambridge South (REC Ref 14/EE/1112).

Note that full information on the approval of the study protocol must also be provided in the manuscript.

Field-specific reporting

Please select the one below that is the best fit for your research. If you are not sure, read the appropriate sections before making your selection.

[x] Life sciences [ ] Behavioural & social sciences [ ] Ecological, evolutionary & environmental sciences

For a reference copy of the document with all sections, see nature.com/documents/nr-reporting-summary-flat.pdf

Life sciences study design

All studies must disclose on these points even when the disclosure is negative.

Sample size	Sample size was determined by the number of participants belonging to each cohort.
Data exclusions	De novo variants with any possibility of duplication between cohorts were conservatively excluded from the study, as described in the methods. No other data were excluded.
Replication	Data were produced using the largest / most highly powered datasets available at present, and can be expanded as cohort sizes increase.
Randomization	Randomisation was not applicable to this study because participants were not subject to any treatment or exposure.
Blinding	Blinding was not possible or applicable for this retrospective analysis.

Reporting for specific materials, systems and methods

We require information from authors about some types of materials, experimental systems and methods used in many studies. Here, indicate whether each material, system or method listed is relevant to your study. If you are not sure if a list item applies to your research, read the appropriate section before selecting a response.

Materials & experimental systems

n/a	Involved in the study
☒	☐ Antibodies
☒	☐ Eukaryotic cell lines
☒	☐ Palaeontology and archaeology
☒	☐ Animals and other organisms
☒	☐ Clinical data
☒	☐ Dual use research of concern
☒	☐ Plants

Methods

n/a	Involved in the study
☒	☐ ChIP-seq
☒	☐ Flow cytometry
☒	☐ MRI-based neuroimaging

Plants

Seed stocks	N/A
Novel plant genotypes	N/A
Authentication	N/A
